# Supplementary material for: Antiproliferative and Antiangiogenic Properties of New VEGFR-2-targeting 2-thioxobenzo[g]quinazoline Derivatives (In Vitro)
Source: Molecules. 2020 Dec 15;25(24):5944. doi: 10.3390/molecules25245944 (PMC7765401; doi:10.3390/molecules25245944)
Supplement: Supplementary file 1 [file molecules-25-05944-s001.pdf]

Supplementary

Table S1: Equations 1–6

$$R^2 = 1 - \frac{\sum(Y_{exp} - Y_{pred})^2}{\sum(Y_{exp} - Y_{mtraining})^2}, \quad (1)$$

$$R^2_{\text{adj}} = 1 - (1 - R^2)\frac{N-1}{N-P-1} = \frac{(N-1)R^2 - P}{N-P+1}, \quad (2)$$

$$Q^2_{cv} = 1 - \frac{\sum(Y_{pred} - Y_{exp})^2}{\sum(Y_{exp} - Y_{mtraining})^2}, \quad (3)$$

$$R^2_{test} = 1 - \frac{\sum(Y_{pred} - Y_{exp})^2}{\sum(YY_{exp} - Y_{mntraining})^2}, \quad (4)$$

HepG2-pIC50 = 4.48354-0.01440 * (PEOE_VSA+5) -0.00635 * (PEOE_VSA-1) -0.01285 * (SMR_VSA1) -0.01289 * (SMR_VSA4) -0.00459 * (SMR_VSA7) +0.01290 * (SlogP_VSA0) +0.00693 * (SlogP_VSA9) +0.17415 * (a_acc) +0.05099 * (a_aro)…..(5)

MCF-7_pIC50 = 3.38856+0.00930 * (PEOE_VSA+0) -0.01461 * (PEOE_VSA+1) +0.02067 * (PEOE_VSA-0) +0.01383 * (PEOE_VSA-1) -0.02555 * (SMR_VSA2) -0.03142 * (SMR_VSA5) -0.00882 * (SMR_VSA6) -0.02123 * (SMR_VSA7) -0.01230 * (SlogP_VSA1) +0.02673 * (SlogP_VSA5) +0.02560 * (SlogP_VSA7) +0.01638 * (SlogP_VSA8) +0.01054 * (SlogP_VSA9) +0.04323 * (TPSA) -0.60955 * (a_acc) +0.14295 * (b_rotN)………………….(6)

Table S2: Minimum recommended values of validated parameters for generally acceptable QSAR

Symbol	Name	Value
R^2	Coefficient of determination	≥ 0.6
Q^2_{cv}	Cross-validation coefficient	≥ 0.5
R^2_{test}	The coefficient of determination for external test set	≥ 0.6
$R^2 - Q^2$	Difference between R^2 and Q^2	≤ 0.3
N_{test}	Minimum number of an external test set	≥ 5

Table S3. Correlation matrix of from PLS analysis for HepG2 variables

	PEOE_VSA+0	PEOE_VSA-1	SMR_VSA1	SMR_VSA4	SMR_VSA7
PEOE_VSA+5	1				
PEOE_VSA-1	0.20	1			
SMR_VSA1	0.46	0.50	1		
SMR_VSA4	0.09	0.13	0.47	1	
SMR_VSA7	0.13	0.43	0.17	0.41	1

Table S4. Correlation matrix of from PLS analysis for MCF7 variables

	PEOE_VSA+0	PEOE_VSA-1	SMR_VSA1	SMR_VSA4	SMR_VSA7
PEOE_VSA+0	1				
PEOE_VSA+1	0.45	1			
PEOE_VSA+0	0.38	0.28	1		
PEOE_VSA-1	0.50	0.01	0.36	1	
SMR_VSA2	0.06	0.1	0.04	0.21	1

Table S5. Chemical structure of 2-thioxo-benzo[g]quinazoline derivatives and their cytotoxicity activities on HepG2 and MCF7

		R1	R2	pIC_{50}(exp) HepG2	pIC_{50} (pred) HepG2	pIC_{50} (exp) MCF7	pIC_{50} (pred) MCF7
**1**				4.394	4.242	4.9706	5.2104263
**2**				4.442	4.465	4.9788	5.0270052
**3**			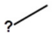	4.524	4.292	4.996	5.084344
**4**				4.458	4.343	4.991	4.944325
**5**				4.504	4.446	4.975	5.120699
**6**				4.541	4.495	4.9706	5.140266
**7**				4.444	4.313	5.0089	5.340398491
**8**				4.504	4.623	4.963	5.211577
**9**				4.499	4.515	4.991	4.900923
**10**				4.441	4.566	5.056	4.760903

**11**		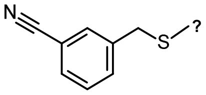	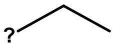	4.466	4.5778	4.975	5.156978
**12**		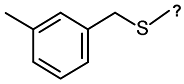	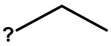	4.513	4.669	4.951	4.937278
**13**		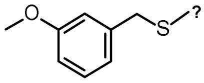	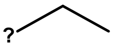	4.561	4.766	4.996	5.028156
**14**		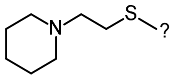	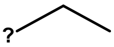	4.558	4.434	5.0177	4.819856
**15**		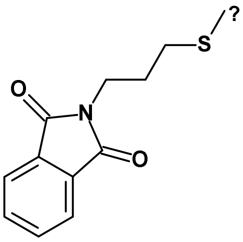	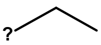	4.553	4.468	5.0269	5.458392
**16**		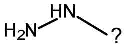	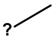	4.539	4.315	4.9830	5.3950586
**17**		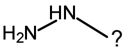	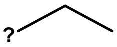	4.526	4.538	4.9957	5.2116375

Table S6: Statistical Validation Results of QSARModel for HepG2 & MCF7 cell line

	Parameter Calculated	
Validation	**HepG2**	MCF7
R^2 (Coefficient of determination)	0.71947	0.85989
root mean square error (RMSE)	0.18169	0.2005
Q_{cv}^2 (Cross-validation coefficient)	0.62709	0.7277
Cross-Validated (RMSE)	0.21027	0.2822
R_{test}^2(The coefficient of determination for external test set)	0.5224	0.6751
Difference between R^2 and Q^2	0.09238	0.13219
Number of an external test set	21	24
Number of Descriptors	11	16
R_{adj}^2	0.64529	0.79599

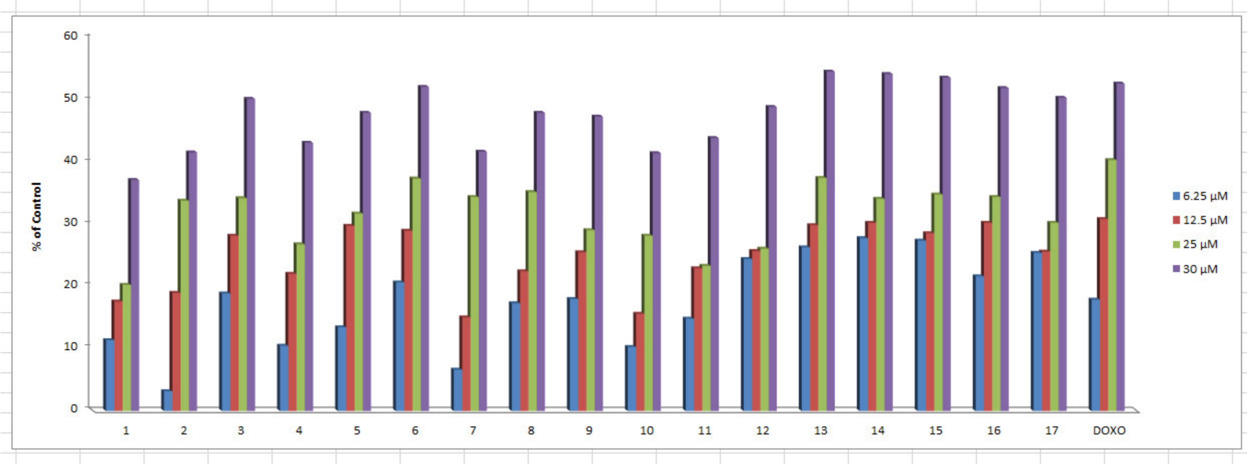

Figure S1: Dose-dependent antiproliferative data of the compounds on the HepG-2 human cancer type, according to the MTT assay after 48 h of exposure.

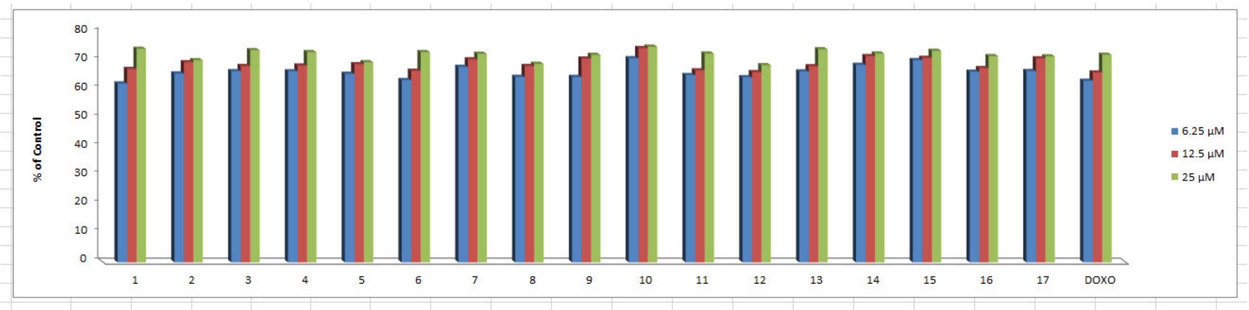

Figure S2: Dose dependent antiproliferative data of the compounds on the MCF-7 human cancer type according to the MTT assay after 48 h of exposure.

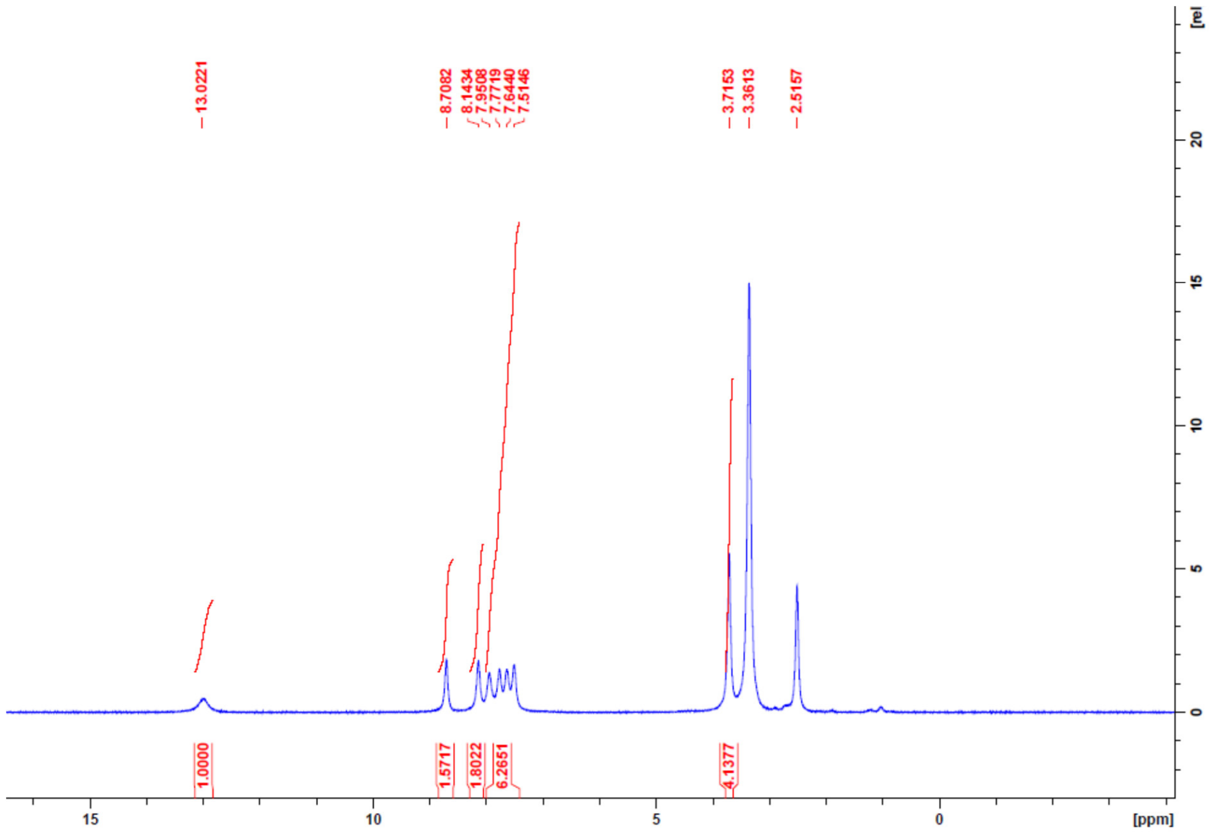

^{1}H-NMR of compound 1

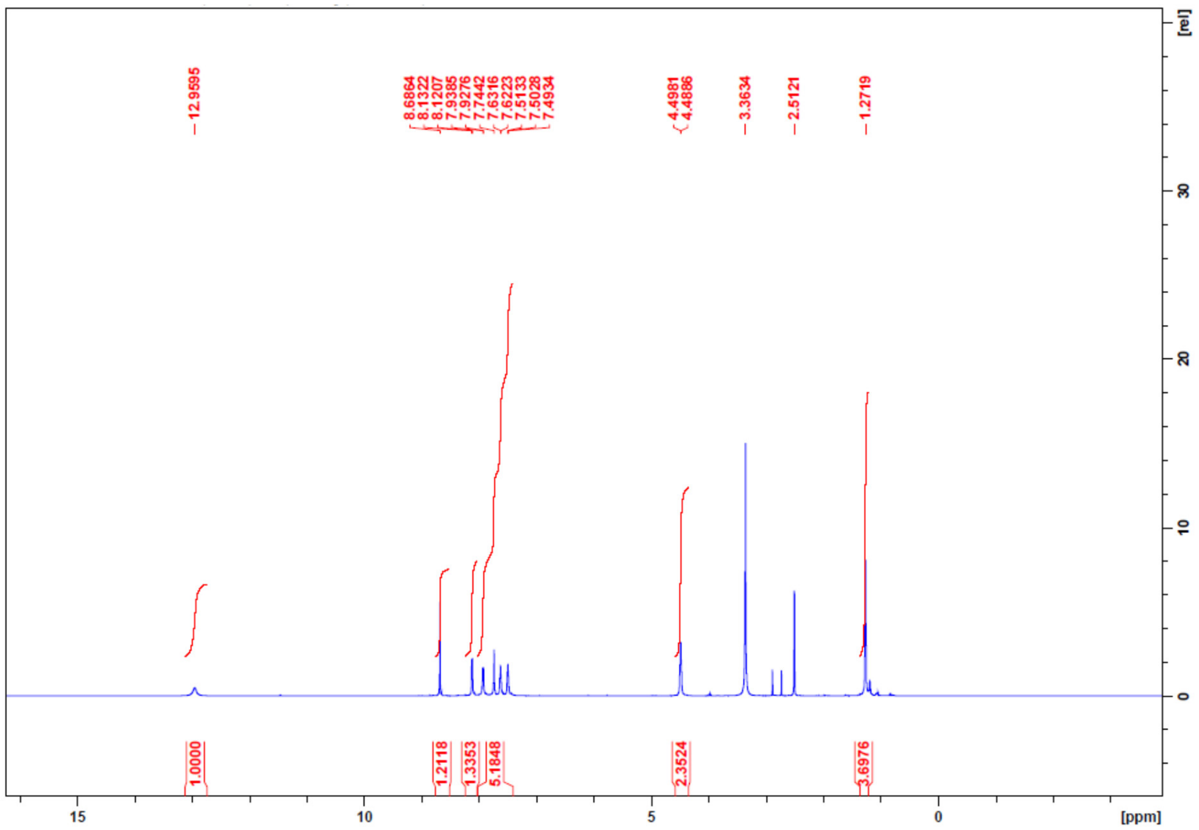

^{1}H-NMR of compound 2

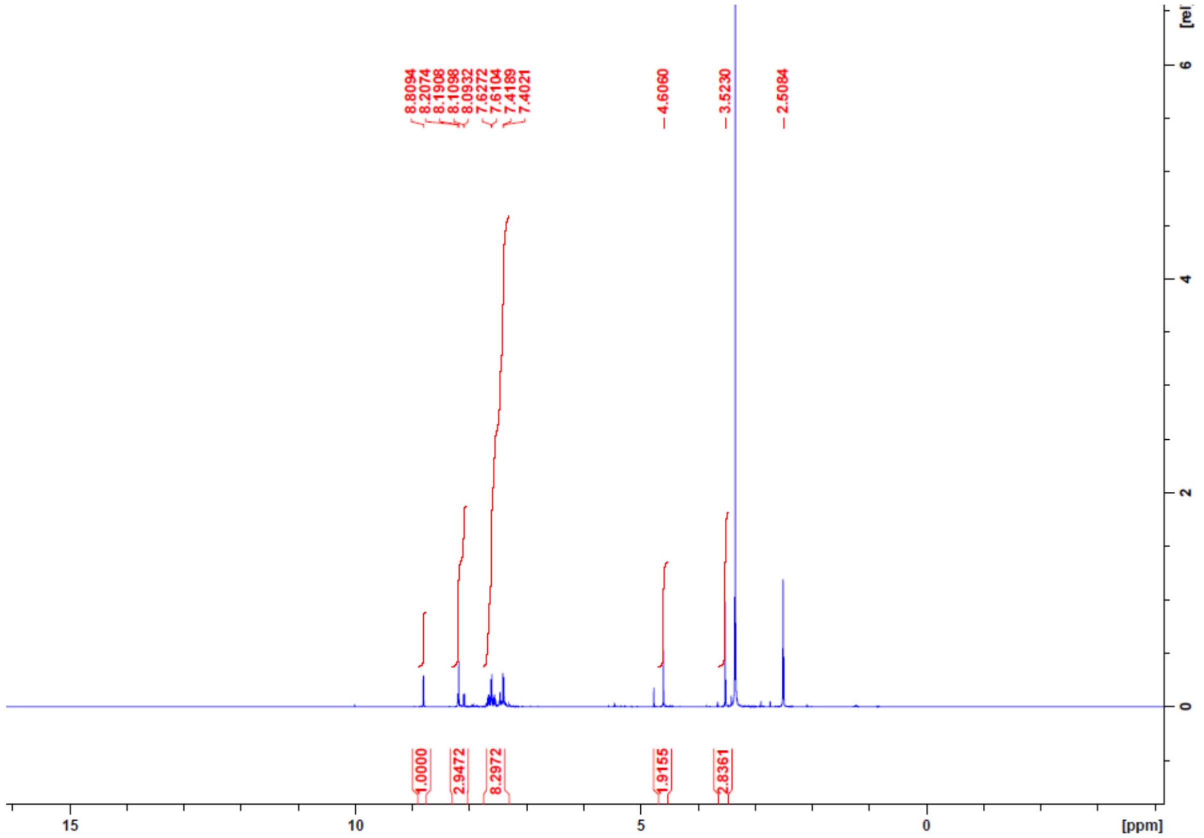

^{1}H-NMR of compound 6

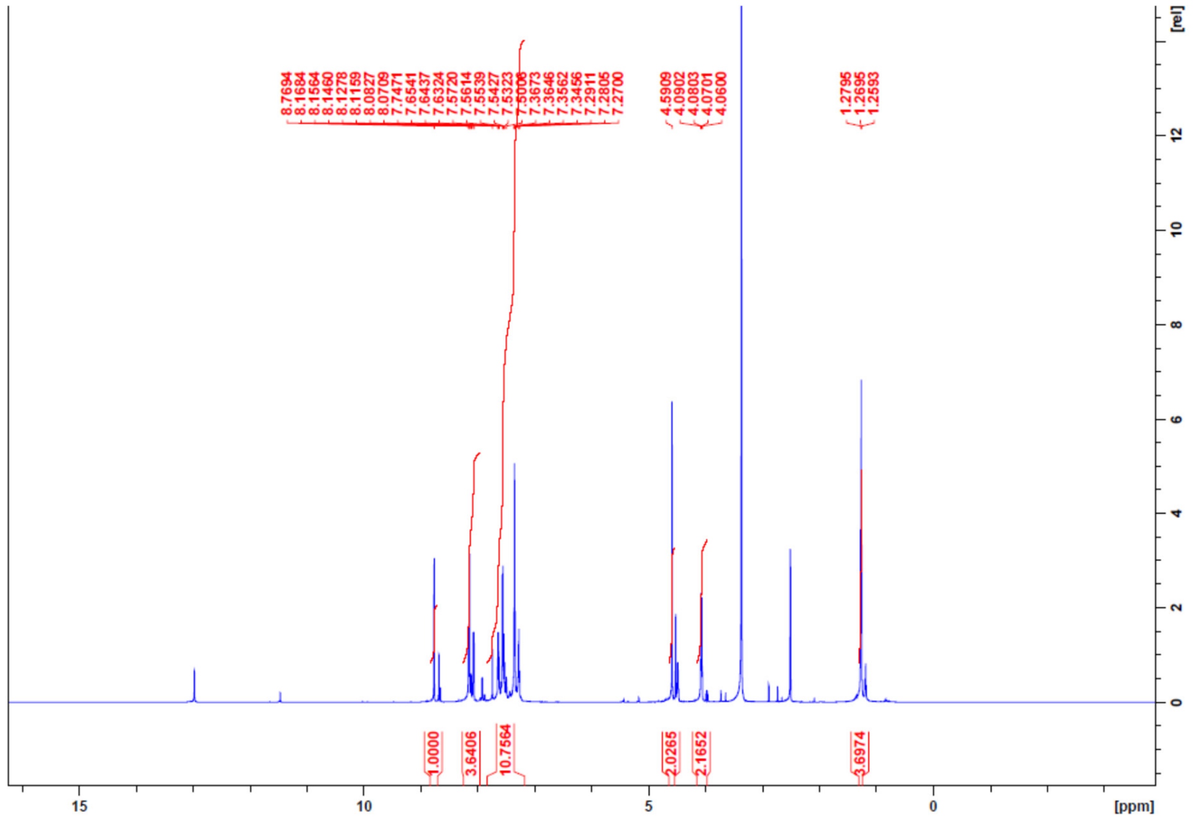

^{1}H-NMR of compound 10

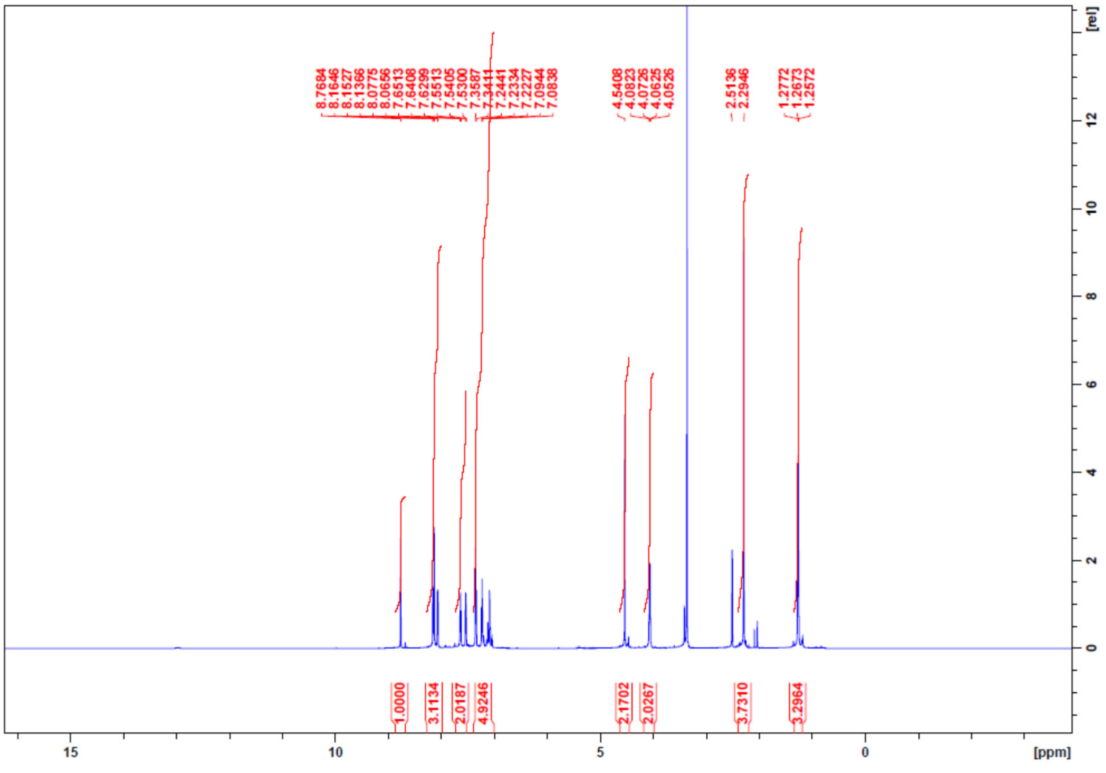

^{1}H-NMR of compound 12

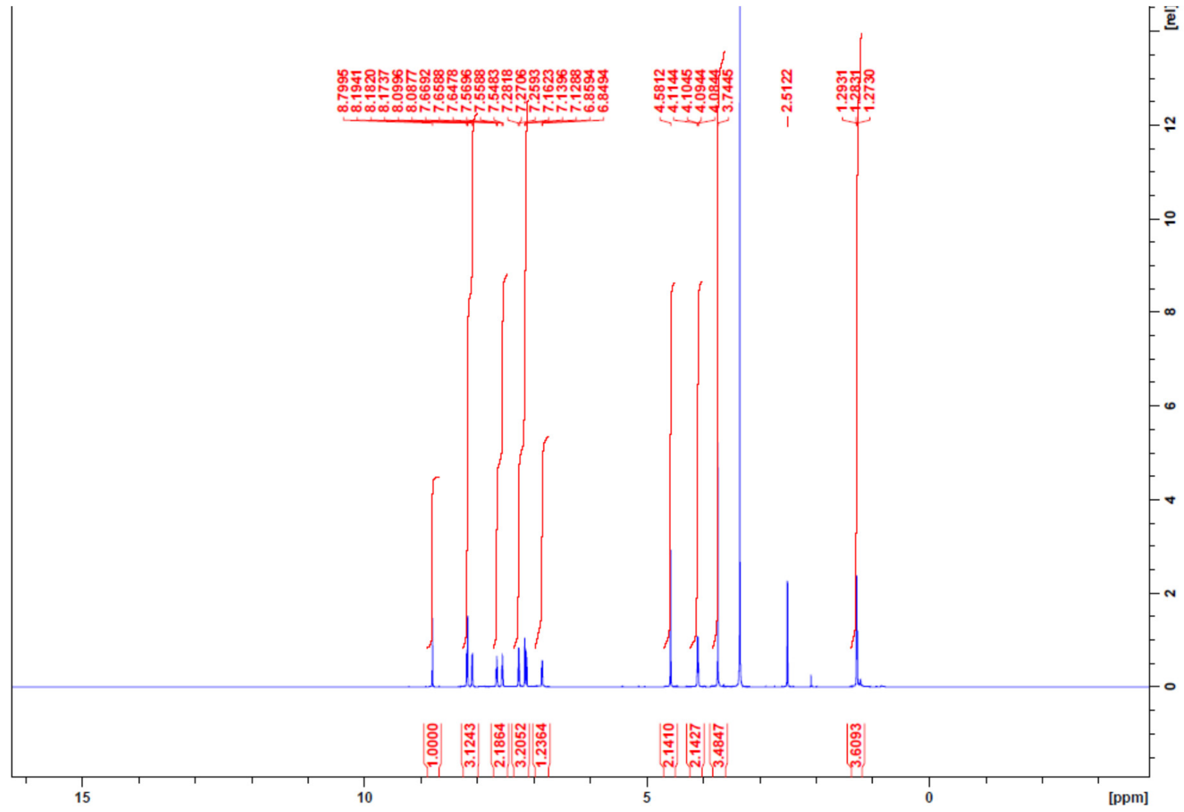

^{1}H-NMR of compound 13

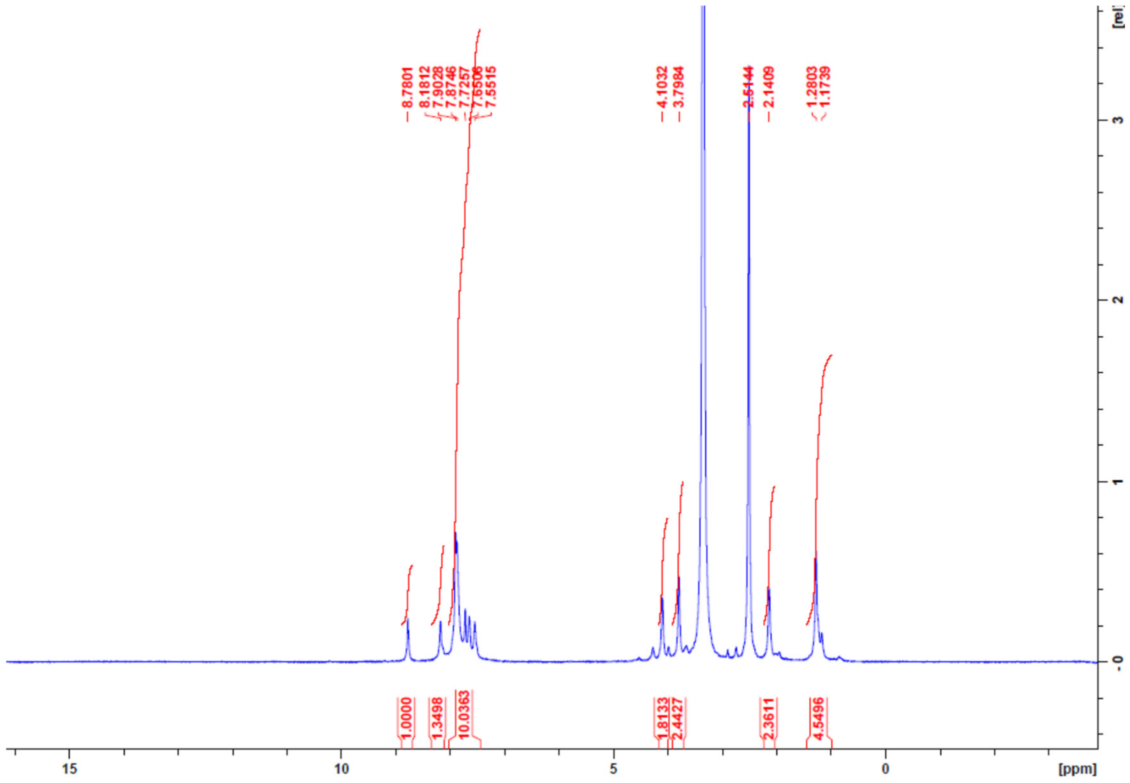

^{1}H-NMR of compound 15

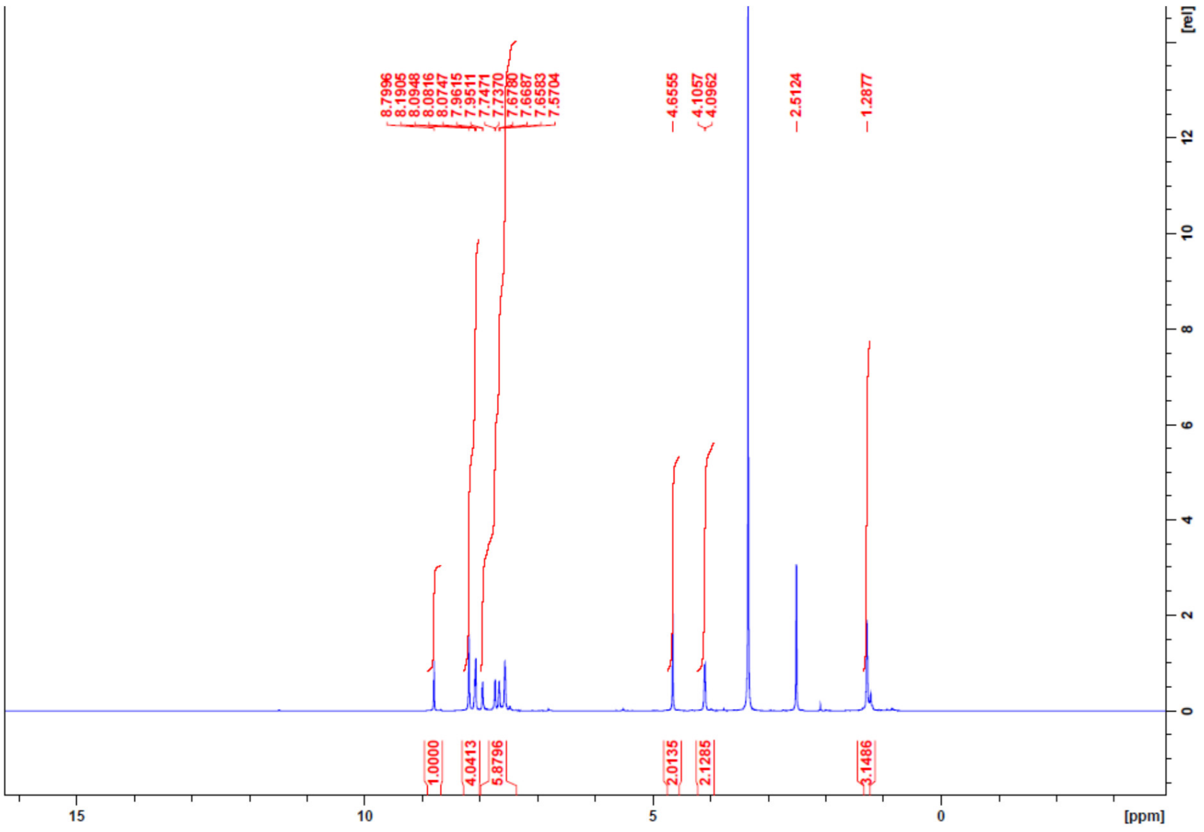

^{1}H-NMR of compound 11

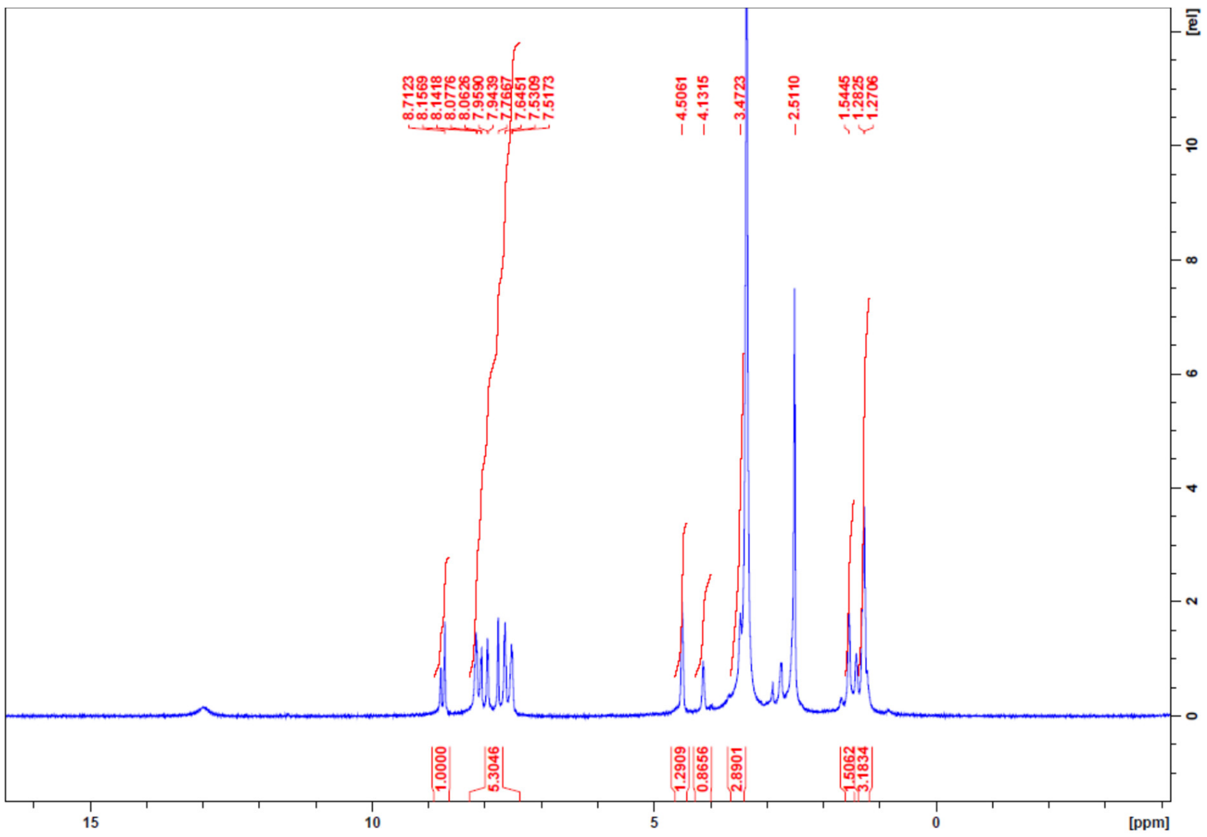

^{1}H-NMR of compound 14

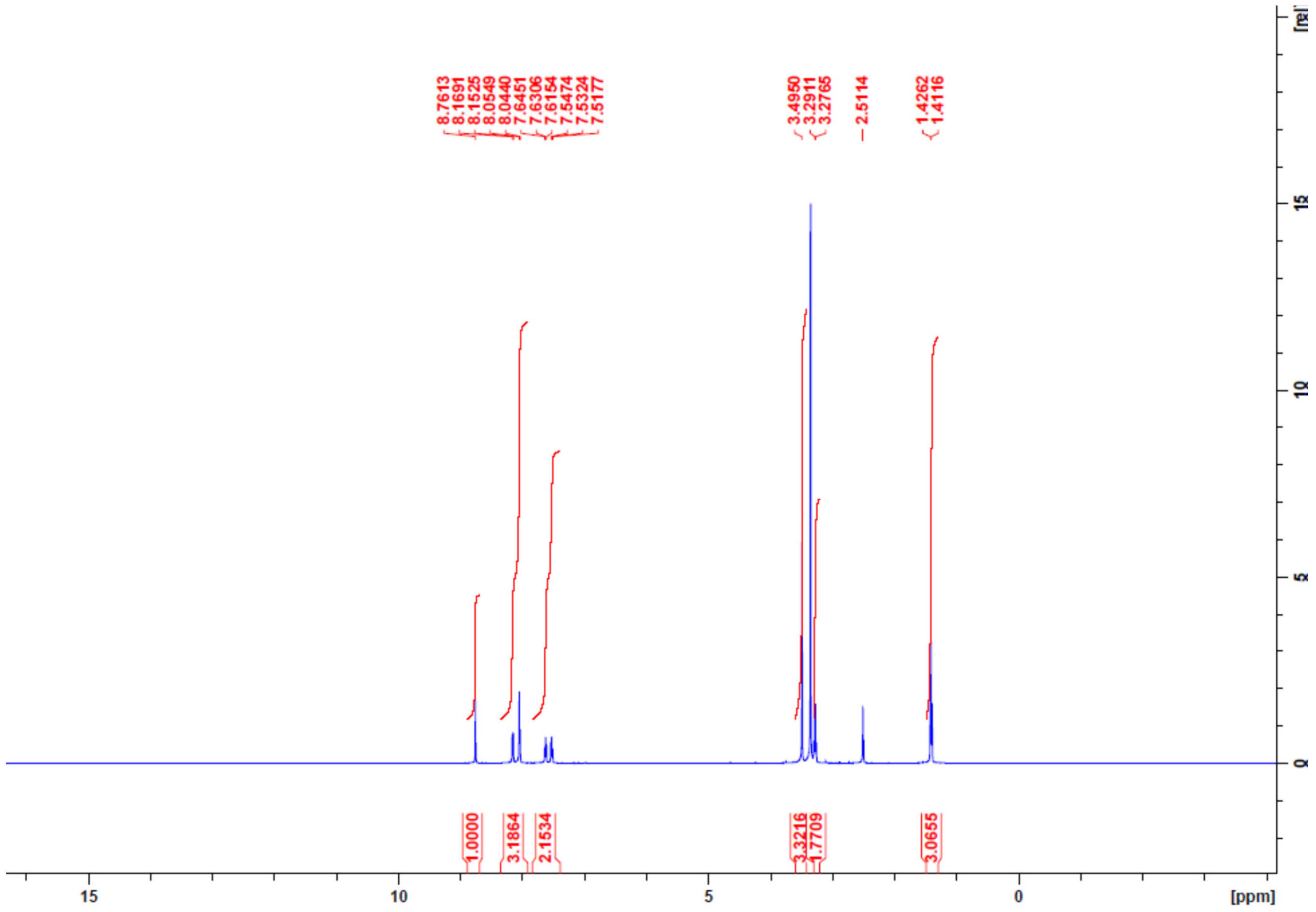

^{1}H-NMR of compound 3

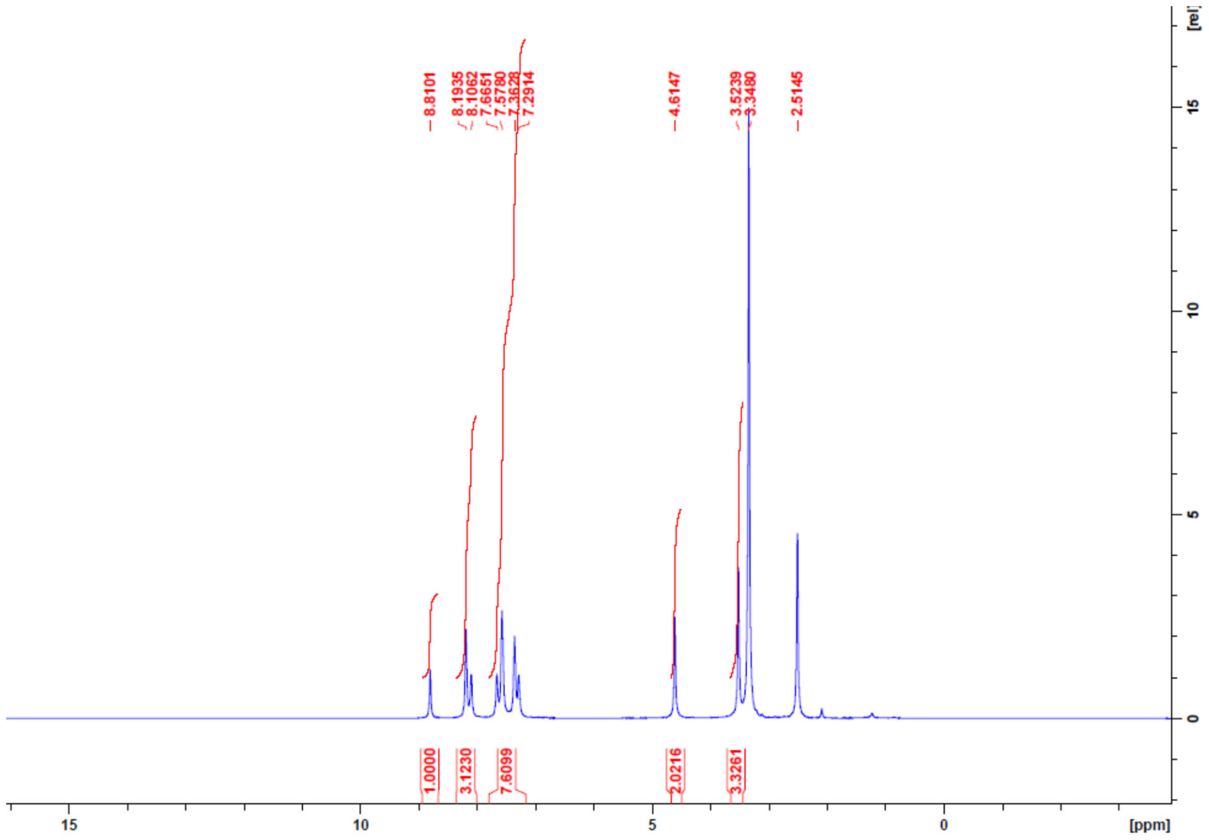

^{1}H-NMR of compound 4

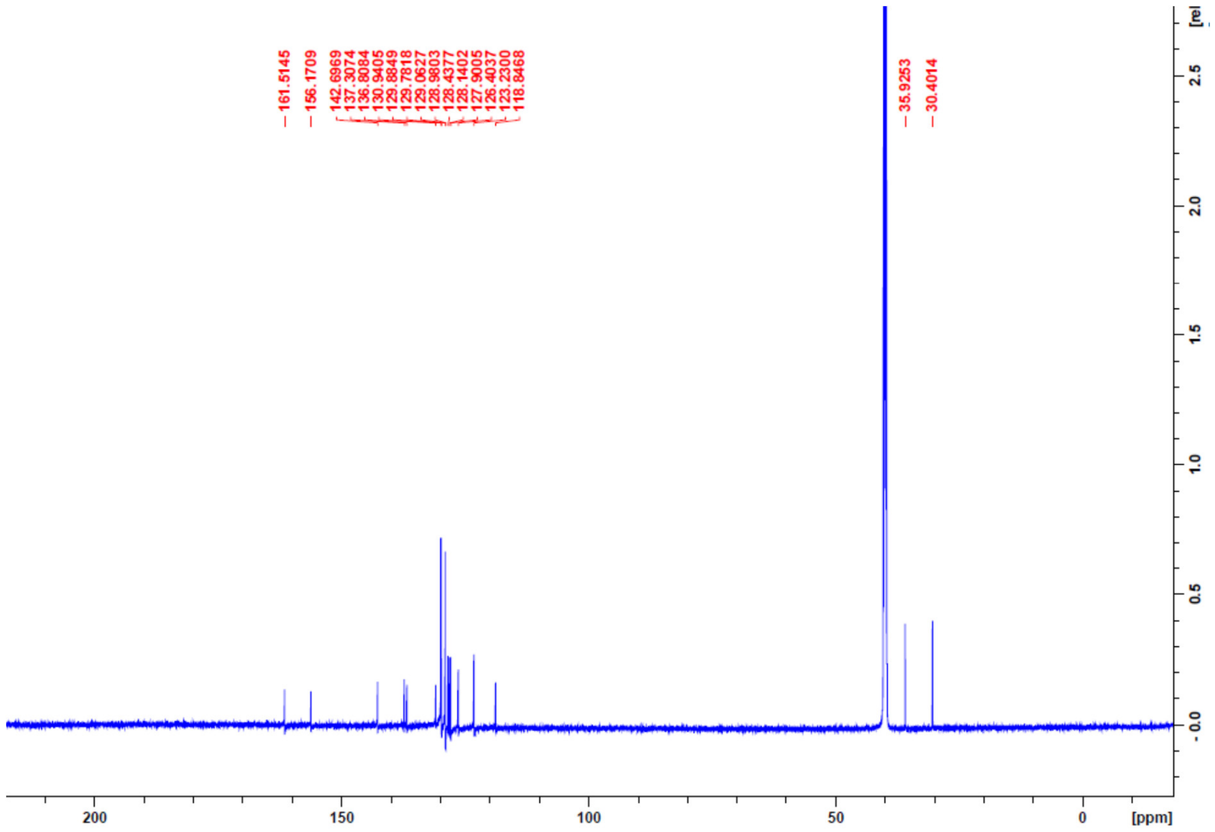

^{13}C-NMR of compound 4

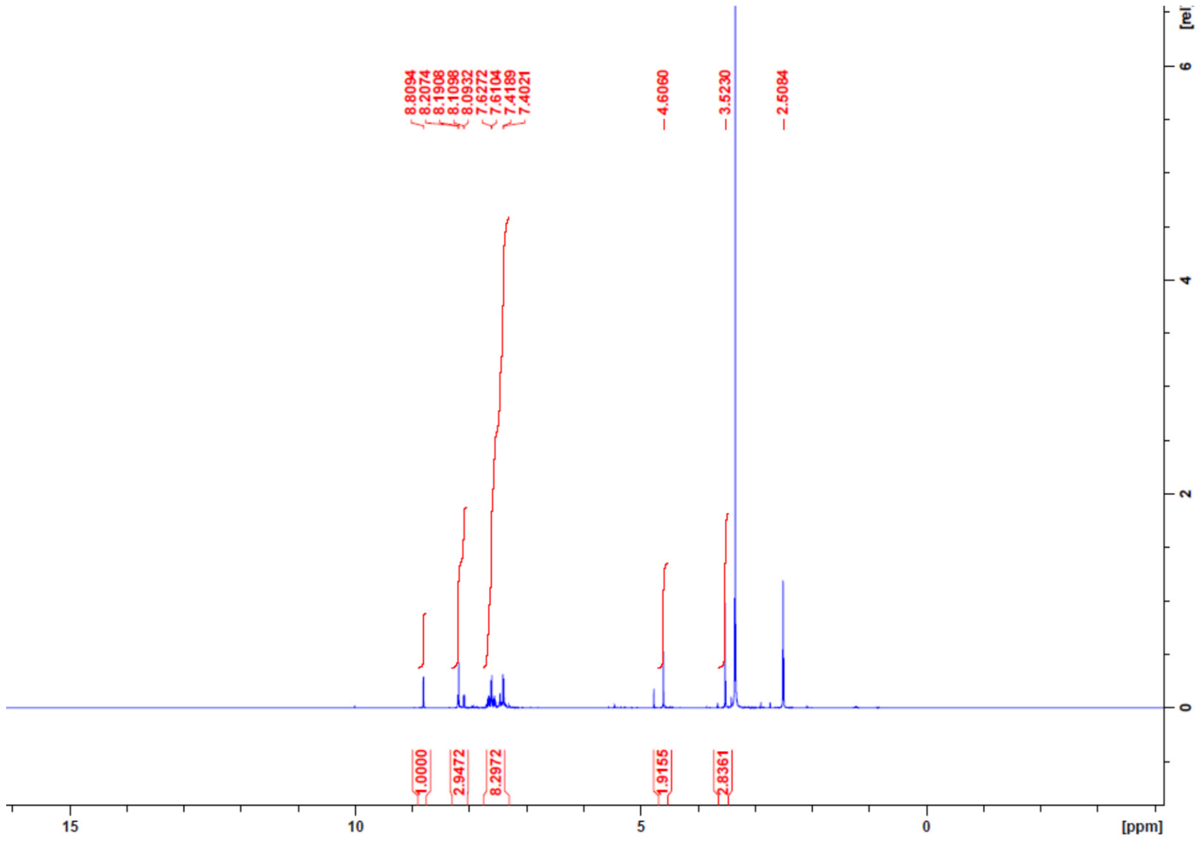

^{1}H-NMR of compound 6

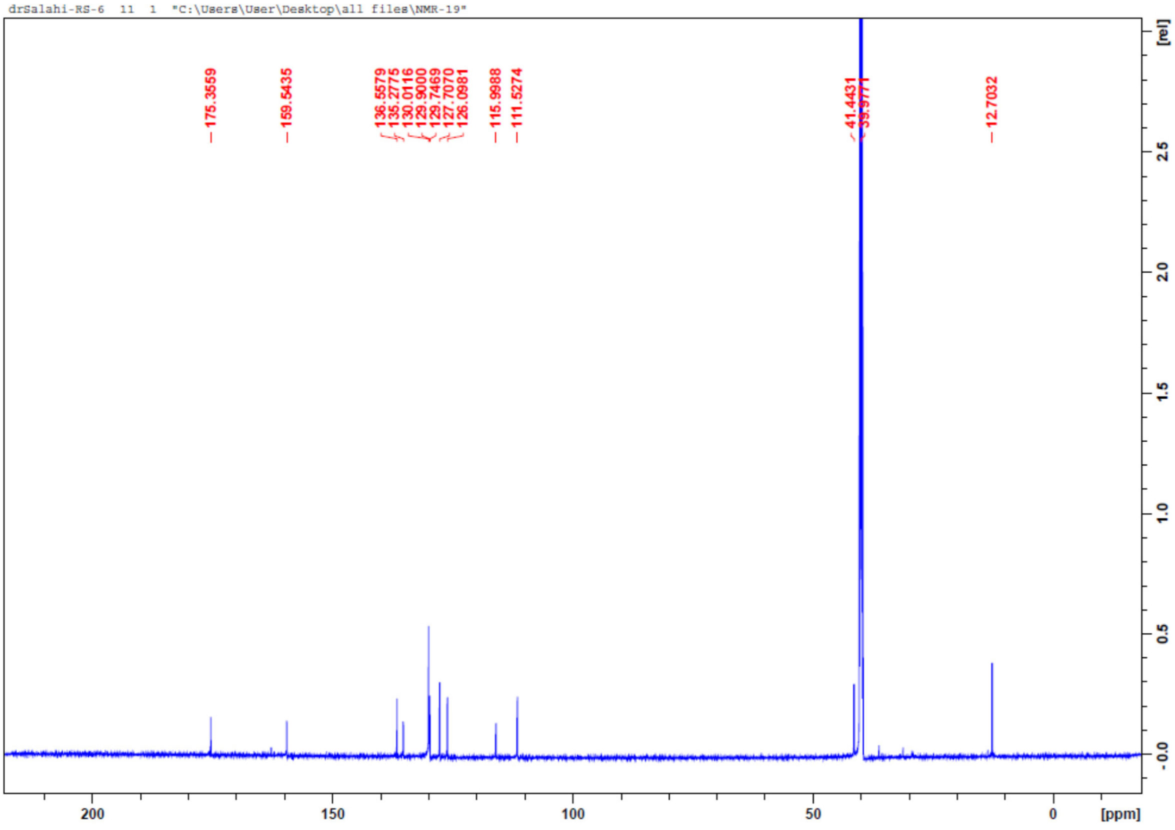

13C-NMR of compound 2

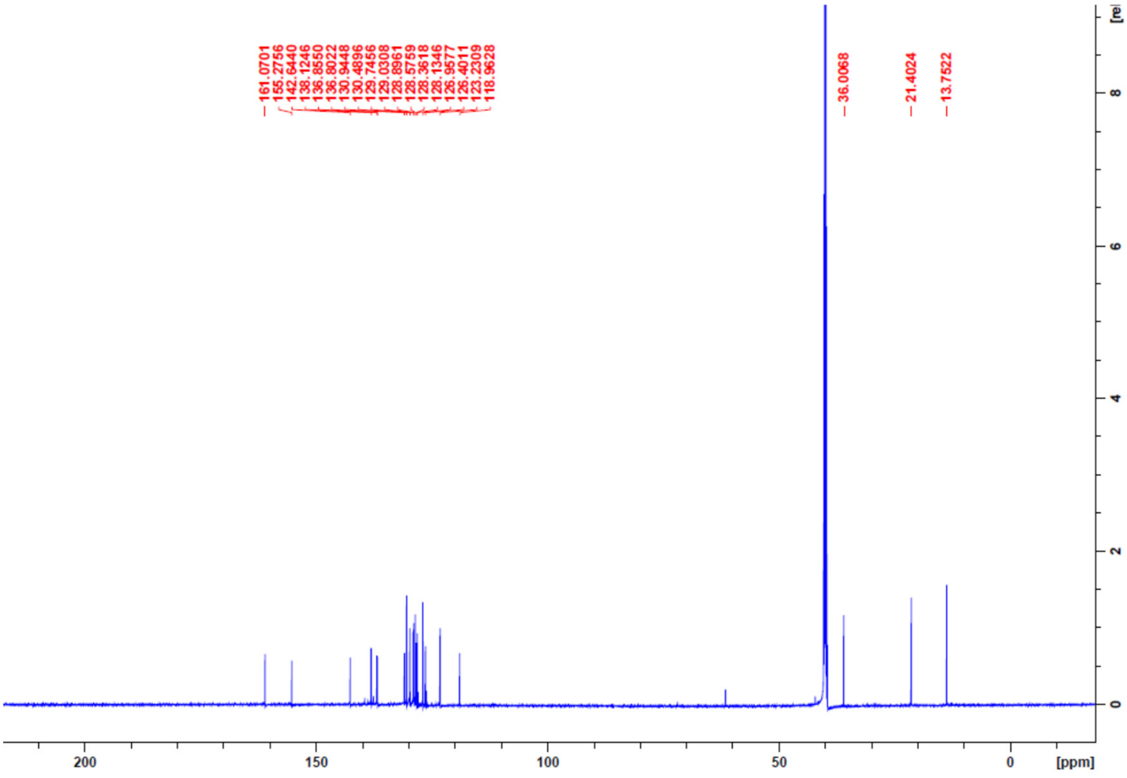

13C-NMR of compound 12

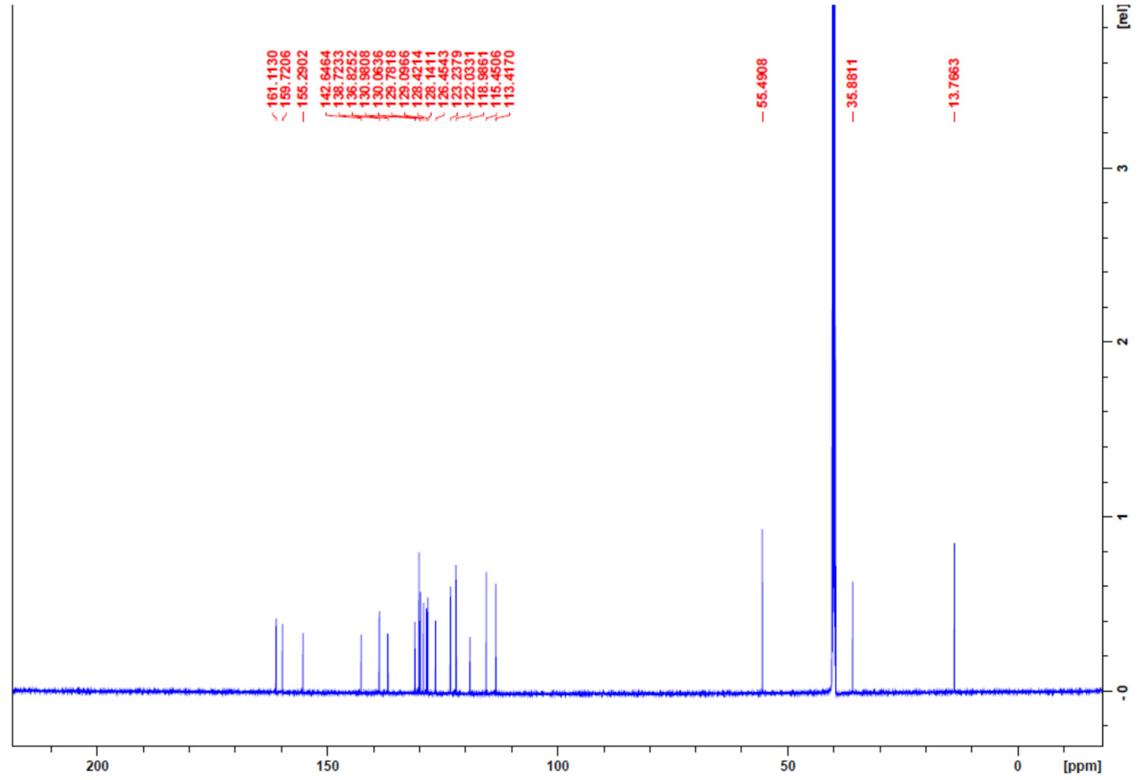

^{13}C-NMR of compound 13

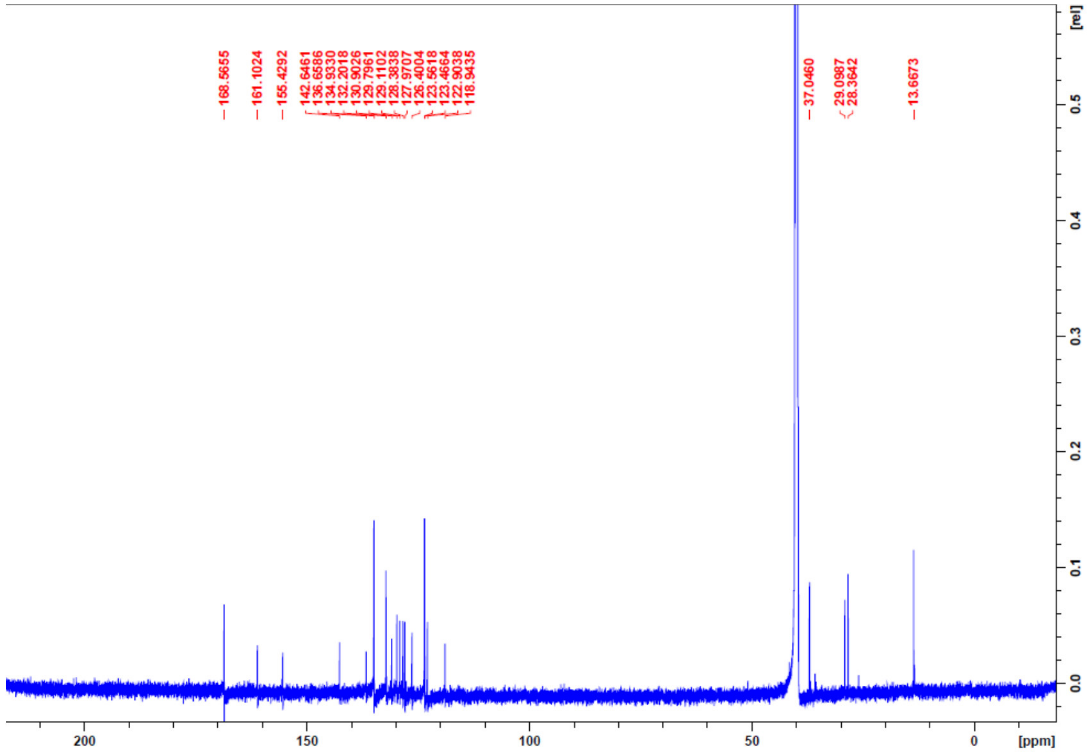

^{13}C-NMR of compound 15

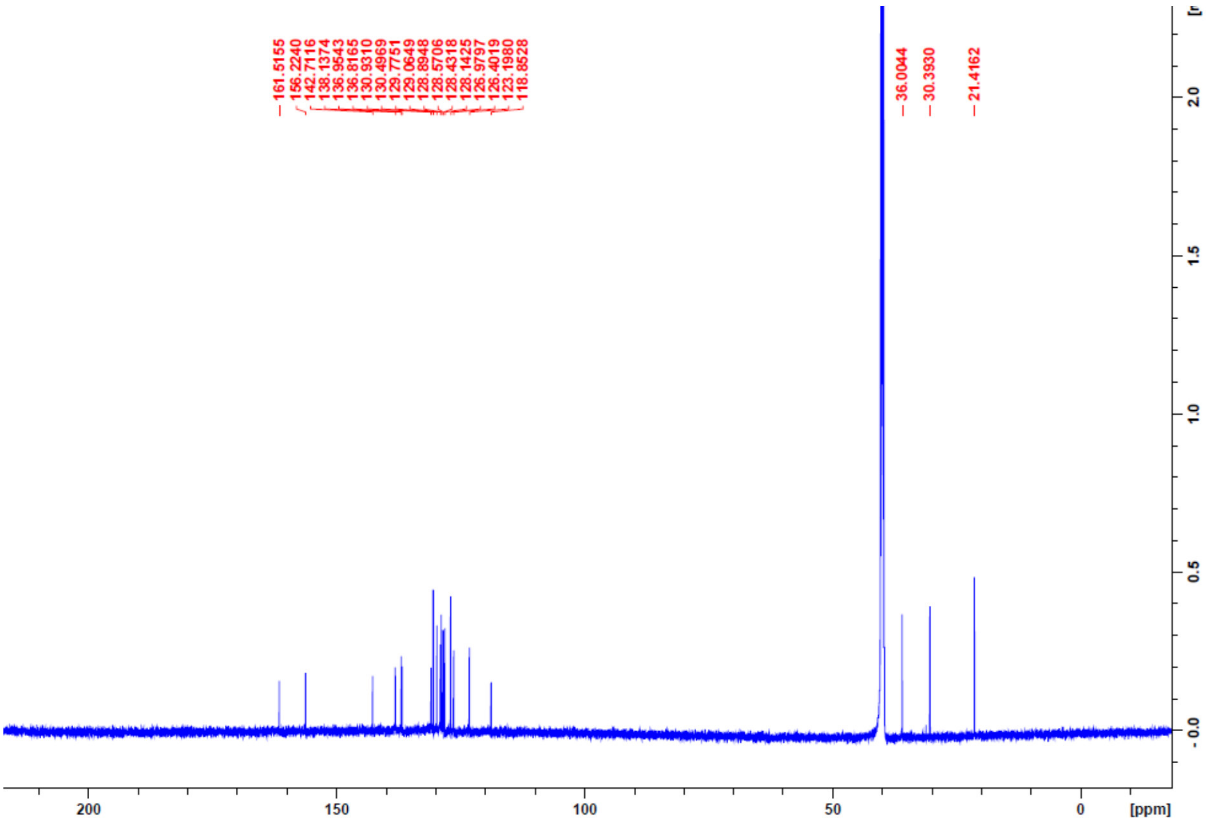

^{13}C-NMR of compound 5

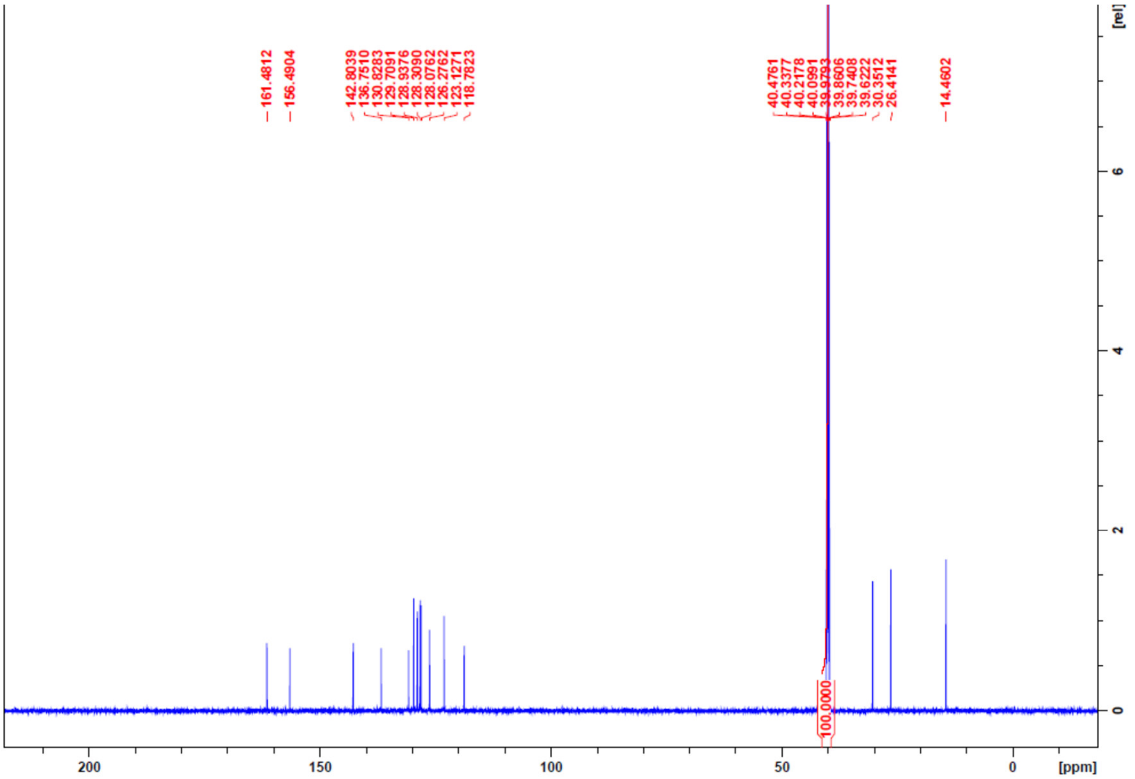

^{13}C-NMR of compound 3
